# The acoustic characteristics of fine crackles predict honeycombing on high-resolution computed tomography

**DOI:** 10.1186/s12890-019-0916-5

**Published:** 2019-08-17

**Authors:** Toshikazu Fukumitsu, Yasushi Obase, Yuji Ishimatsu, Shota Nakashima, Hiroshi Ishimoto, Noriho Sakamoto, Kosei Nishitsuji, Shunpei Shiwa, Tomoya Sakai, Sueharu Miyahara, Kazuto Ashizawa, Hiroshi Mukae, Ryo Kozu

**Affiliations:** 1 Department of Cardiopulmonary Rehabilitation Science, Nagasaki University Graduate School of Biomedical Sciences, 1-7-1 Sakamoto, Nagasaki, 852-8520 Japan; 2 Department of Respiratory Medicine, Nagasaki University Graduate School of Biomedical Sciences, 1-7-1 Sakamoto, Nagasaki, 852-8501 Japan; 3 Department of Nursing, Nagasaki University Graduate School of Biomedical Sciences, 1-7-1 Sakamoto, Nagasaki, 852-8520 Japan; 4 Nagasaki University Graduate School of Engineering, 1-14 Bunkyo, Nagasaki, 852-8521 Japan; 5 Department of Clinical Oncology, Nagasaki University Graduate School of Biomedical Sciences, 1-7-1 Sakamoto, Nagasaki, 852-8501 Japan

**Keywords:** Fine crackles, Lung sounds, Onset timing, Frequency, Time-expanded waveform analysis, Honeycombing, Pulmonary fibrosis

## Abstract

**Background:**

Honeycombing on high-resolution computed tomography (HRCT) is a distinguishing feature of usual interstitial pneumonia and predictive of poor outcome in interstitial lung diseases (ILDs). Although fine crackles are common in ILD patients, the relationship between their acoustic features and honeycombing on HRCT has not been well characterized.

**Methods:**

Lung sounds were digitally recorded from 71 patients with fine crackles and ILD findings on chest HRCT. Lung sounds were analyzed by fast Fourier analysis using a sound spectrometer (Easy-LSA; Fukuoka, Japan). The relationships between the acoustic features of fine crackles in inspiration phases (onset timing, number, frequency parameters, and time-expanded waveform parameters) and honeycombing in HRCT were investigated using multivariate logistic regression analysis.

**Results:**

On analysis, the presence of honeycombing on HRCT was independently associated with onset timing (early vs. not early period; odds ratios [OR] 10.407, 95% confidence interval [95% CI] 1.366–79.298, *P* = 0.024), F99 value (the percentile frequency below which 99% of the total signal power is accumulated) (unit Hz = 100; OR 5.953, 95% CI 1.221–28.317, *P* = 0.029), and number of fine crackles in the inspiratory phase (unit number = 5; OR 4.256, 95% CI 1.098–16.507, *P* = 0.036). In the receiver-operating characteristic curves for number of crackles and F99 value, the cutoff levels for predicting the presence of honeycombing on HRCT were calculated as 13.2 (area under the curve [AUC], 0.913; sensitivity, 95.8%; specificity, 75.6%) and 752 Hz (AUC, 0.911; sensitivity, 91.7%; specificity, 85.2%), respectively. The multivariate logistic regression analysis additionally using these cutoff values revealed an independent association of number of fine crackles in the inspiratory phase, F99 value, and onset timing with the presence of honeycombing (OR 33.907, 95% CI 2.576–446.337, *P* = 0.007; OR 19.397, 95% CI 2.311–162.813, *P* = 0.006; and OR 12.383, 95% CI 1.443–106.293, *P* = 0.022; respectively).

**Conclusions:**

The acoustic properties of fine crackles distinguish the honeycombing from the non-honeycombing group. Furthermore, onset timing, number of crackles in the inspiratory phase, and F99 value of fine crackles were independently associated with the presence of honeycombing on HRCT. Thus, auscultation routinely performed in clinical settings combined with a respiratory sound analysis may be predictive of the presence of honeycombing on HRCT.

## Background

Honeycombing on high-resolution computed tomography (HRCT) is a distinguishing feature of usual interstitial pneumonia (UIP), the hallmark of idiopathic pulmonary fibrosis (IPF), and must be present for a definite HRCT diagnosis of UIP to be made. Radiological and pathological UIP diagnoses are reported to show concordances of 90–100%. Accordingly, the ATS/ERS/JRS/ALAT clinical practice guideline for IPF diagnosis 2018 recommends *NOT* performing a surgical lung biopsy (SLB) in patients with an HRCT pattern of UIP, while it suggests performing SLB (conditional) in patients with an HRCT pattern of probable UIP, indeterminate UIP, and alternative diagnosis of UIP [1]. Furthermore, honeycombing on HRCT is predictive of a poor outcome not only in IPF but also in nonspecific interstitial pneumonia and connective tissue disease-related fibrotic interstitial lung disease (ILD) [2–4].

Fine crackles are heard in 60% of patients with interstitial pneumonia [5] as well as in 100% of patients with honeycombing on HRCT and a UIP pattern [6]. Recently, Sgalla et al. reported that fine crackles (also called Velcro crackles) could predict the presence of fibrotic ILD according to a study in subjects undergoing chest HRCT scans for various clinical indications [7]. Moreover, Cottin et al. advise that physicians should perform chest auscultation and when bilateral fine crackles are heard, conduct an examination of the chest radiograph and/or CT image to detect IPF earlier [8].

There are only a few reports investigating the relationship between clinical or radiological findings and acoustic variables. In patients with fine crackles, time and frequency parameters in the time-expanded waveform analysis (e.g., two-cycle duration [2CD], initial deflection width [IDW], and largest deflection width [LDW]) are frequently employed, although the Computerized Respiratory Sound Analysis (CORSA) guidelines recommend the additional use of number and timing for the analysis of fine crackles [9]. In the present study, we provide a comprehensive analysis of the acoustic characteristics of fine crackles in patients with honeycombing compared to those without honeycombing and investigate whether these acoustic parameters can predict the presence of honeycombing on HRCT.

## Methods

### Study setting and subjects

This study included 71 patients with ILD who received medical care at Nagasaki University Hospital (Nagasaki, Japan) between July 2015 and June 2017. All participants presented at their bilateral lower back area fine crackles as determined by chest auscultation by at least two chest physicians and underwent HRCT chest scans for various clinical indications. Exclusion criteria were severe dyspnea, an oxygen saturation < 90% during the lung sound recording, and comorbid conditions (e.g., neurological impairment, severe cognitive impairment, and pregnancy) affecting the recording of respiratory sounds. The HRCT features relating to ILD, independently reviewed by two chest physicians and one radiologist, were as follows: honeycombing, traction bronchiectasis, reticulation, and ground-glass opacity. The study protocol was approved by the Human Ethics Review Committee at Nagasaki University Hospital, and all participants provided written informed consent before enrolment.

### Examination and assessment of HRCT

Whole-lung CT images were obtained with two 64-detector row CT scanners (Aquilion 64, Toshiba Medical Systems, Otawara, Tochigi, Japan or Somatom Definition, Siemens Healthcare, Erlangen, Germany) using the following settings: 1.0-mm section width with 1.0-mm reconstruction interval, beam pitch 0.828, tube voltage 120 kVp, tube current volume EC, SD11 (Toshiba); beam pitch 0.9, tube voltage 120 kVp, 100–200 mA (Siemens). The images were photographed at the lung parenchymal window setting (window width 1600 HU, window level − 600 HU). Two chest physicians and one radiologist (with 23, 26, and 32 years of experience in chest CT interpretation) who were blinded to the clinical data of the patients independently performed CT findings evaluations including that for honeycombing, traction bronchiectasis, and ground-glass opacities, as defined by the ATS/ERS/JRS/ALAT clinical practice guideline for IPF diagnosis 2018 [1].

### Collection of lung sounds

Lung sounds were recorded at two sites over the bilateral posterior base (at 6 cm below the inferior angle of the scapula and 4 cm from the paravertebral line), where we could most clearly recognize fine crackles using a hand-held stethoscope. The subjects followed onscreen instructions to breathe deeply for approximately 30 s (eight breaths; one breath consists of 2 s-inspiration and 2 s-expiration) to record the lung sounds over each side. The recording system comprised air-coupled microphones, amplifiers, an analog-to-digital converter (UA-4FX; Shizuoka, Japan), and a personal computer.

### Lung sounds analysis

**Fig. 1:**
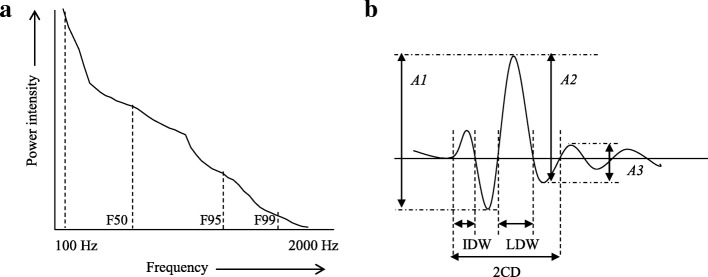
Definition of fine crackles variables. **a** Power spectrum. The percentile frequencies F99, F95, and F50 below which 99, 95, and 50% of the total signal power are respectively accumulated were measured. **b** Time-expanded waveform of crackles. The waveform analysis was used to evaluate the predefined features of fine crackles known as two-cycle duration (2CD), initial deflection width (IDW), largest deflection width (LDW), and amplitude ratios (A2/A1, A3/A1)

The lung sounds acquired by the microphones were amplified and subsequently digitized with 16-bit resolution at a sampling frequency of 44.1 kHz per channel. The lung sounds in the inspiratory phase were analyzed by fast Fourier analysis using a sound spectrometer (Easy-LSA; Fukuoka, Japan) as described previously [10, 11]. The recording system was calibrated using a reference sound pressure (1 kHz; 94 dB [0 dB, 20 mPa]). In order to reduce ambient background noise, the bandwidth for breath sounds was set from 100 to 2000 Hz. The sounds were displayed as a spectrogram, with frequency in Hz on the vertical axis and time on the horizontal axis. The parameters for the sound spectrum were determined by one point of the maximum frequency. The power spectrum (power intensity, with respect to frequency in Hz) is shown in Fig. 1a. The data were automatically calculated using the Easy LSA. The percentile frequencies F99, F95, and F50 below which 99, 95, and 50% of the total signal power are respectively accumulated were measured according to previous reports [12, 13]. The time-expanded waveform analysis was also used to evaluate the predefined features of fine crackles known as 2CD, IDW, LDW [14], and amplitude ratios (A2/A1, A3/A1; Fig. 1b). In accordance with the CORSA guidelines [9], a fine crackle was defined in this study by 2CD < 10 ms. The number of fine crackles was expressed as the average number per inspiratory phase, and the onset timing of fine crackles was divided into three groups (early, mid, and late period of the inspiratory phase).

### Statistical analysis

Continuous variables were expressed as the median with interquartile range. Categorical variables were summarized by frequency. Nominal or ordinal data were contrasted using a Chi-squared test. The Mann-Whitney U test was performed to compare the two ILD groups, with or without honeycombing. Interobserver agreement was determined using Gwet’s AC1 statistics. The agreement by AC1 was assessed as: 0.00–0.20, poor; 0.21–0.40, fair; 0.41–0.60, moderate; 0.61–0.80, good; and 0.81–1.00, very good. Univariate and multivariate logistic regression analyses were performed to identify the acoustic features of fine crackles in the honeycombing group to distinguish them from those in the non-honeycombing group. Multivariate logistic regression analysis was performed using significant variables chosen based on the univariate logistic regression analysis. If a category contained several strongly correlated variables (e.g., the frequency values), the most representative one (e.g., F99) was used in the multivariate logistic regression analysis. With regard to the continuous variables extracted by multivariate logistic regression analysis (number of fine crackles and their F99 value), the upper left corner coordinate point of the receiver operating characteristic (ROC) curve was used to determine the optimal cutoff level for the discrimination between the presence and absence of honeycombing in HRCT. Statistical analyses were performed using the statistical software package SPSS (IBM SPSS Statistics version 21.0; Chicago, IL, USA) and R version 3. 5. 0 (2018-04-23) [15]. The level of significance was set at 0.05.

## Results

### Characteristics of the study groups

The clinical, physiological, and radiological characteristics of the 24 patients with honeycombing (honeycombing group) and the 47 patients without honeycombing (non-honeycombing group) are summarized in Table 1. The interobserver agreement on the presence of honeycombing was substantial (AC1 = 0.88, 95% confidence interval [CI], 0.79–0.97). The median age in the honeycombing group (77.0 years) was higher than that in the non-honeycombing group (67.5 years). The honeycombing group presented less frequently a radiological sign of ground-glass opacity. There were no significant differences in sex, body mass index, lung function, partial pressure of arterial oxygen (PaO_2_), oxygen therapy, and biomarkers of ILD between these two groups.

**Table 1:** Characteristics of patients with interstitial lung disease with or without honeycombing

	Honeycombing group	Non-honeycombing group	*P*
	(*n* = 24)	(*n* = 47)	
Age (y)	77.0 [69.0–79.0]	67.5 [58.2–74.5]	0.116
Male (%)	13 (54.2)	31 (65.9)	0.336
Body mass index (kg/m^2^)	23.1 [21.1–24.9]	25.1 [21.4–28.5]	0.951
Radiological findings			
Traction bronchiectasis	15	27	0.684
Reticulation	18	34	0.812
Ground-glass opacity	12	42	< 0.001
Lung function			
%VC	77.5 [65.2–93.7]	74.8 [65.5–85.2]	0.728
%DL_CO_	62.0 [43.0–70.8]	52.6 [40.9–65.6]	0.258
Oxygen therapy (%)	6 (25.0)	8 (17.0)	0.427
Laboratory data			
PaO_2_ (torr)	83.1 [73.2–85.7]	75.2 [72.4–90.2]	0.781
KL-6 (U/mL)	1509 [780–2385]	1286 [638–2484]	0.149
SpD (ng/mL)	147.0 [85.7–321.0]	271.0[104.8–361.8]	0.931
SpA (ng/mL)	70.4 [47.7–121.5]	81.7 [51.0–103.8]	0.185

Data are presented as the median [25th–75th percentile] or as the number of events (percentage). *VC* Vital capacity, *DL*_*CO*_ Diffusing capacity of the lungs for carbon monoxide, *PaO*_*2*_ Partial pressure of arterial oxygen, *KL-6* Krebs von der Lungen-6*, SpD* Surfactant protein D*, SpA* Surfactant protein A

### Acoustic features of fine crackles in the two study groups

The comparison of the acoustic characteristics revealed significant differences in the onset timing, number, frequency parameters (F99 and F95), and time-expanded waveform parameters (2CD and LDW) of fine crackles (Table 2). Fine crackles began mostly in the early but never in the late period in the honeycombing group, whereas in the non-honeycombing group, they began nearly evenly in all three periods. The number of fine crackles per inspiratory phase was significantly elevated in the honeycombing group compared to the non-honeycombing group (21.5 versus 8.0, *P* < 0.001). The frequency parameters (F99 and F95) were significantly higher in the honeycombing than in the non-honeycombing group (F99, 841 versus 689 Hz, *P* = 0.001; and F95, 527 versus 411 Hz, *P* = 0.003). This means that the fine crackles in the honeycombing group had a higher sound pitch in comparison to the non-honeycombing group. The duration times of 2CD and LDW were shorter in the honeycombing than in the non-honeycombing group (2CD, 5.5 versus 7.0 ms, *P* = 0.046; LDW, 1.8 versus 2.0 ms, *P* = 0.004). There were no significant differences in the amplitude ratios (A2/A1, A3/A1).

**Table 2:** Comparison of the fine crackle characteristics in the honeycombing and the non-honeycombing groups

	Honeycombing	Non-honeycombing	*P*
	(*n* = 24)	(*n* = 47)	
Onset timing			
Early / Mid / Late	20 / 4 / 0	12 / 27 / 8	< 0.001
Number of crackles			
Total (inspiratory phase)	21.5 [14.7–30.3]	8.0 [3.3–13.7]	< 0.001
Early onset timing	23.8 [16.1–30.8]	17.5 [11.8–20.0]	0.026
Mid onset timing	12.8 [8.5–16.3]	6.3 [3.0–9.7]	0.099
Late onset timing	–	3.0 [2.0–7.0]	–
Frequency (Hz)			
F99	841 [782–870]	689 [674–821]	0.001
F95	527 [442–679]	411 [362–457]	0.003
F50	259 [233–289]	237 [220–267]	0.052
Waveform (ms)			
2CD	5.5 [5.4–6.4]	7.0 [5.5–7.8]	0.046
IDW	1.1 [1.0–1.5]	1.5 [1.0–1.5]	0.362
LDW	1.8 [1.5–2.0]	2.0 [2.0–2.5]	0.004
Amplitude			
A_2_/A_1_	0.75 [0.68–0.81]	0.75 [0.62–0.84]	0.553
A_3_/A_1_	0.30 [0.22–0.38]	0.27 [0.20–0.41]	0.344

Data are presented as the median [25th–75th percentile] or as the number of events. *F50, F95,* and *F99* Frequency at 50, 95, and 99% of the total signal power, *2CD* Two-cycle duration, *IDW* Initial deflection width, *LDW* Largest deflection width

### Associations between acoustic features of fine crackles and honeycombing in HRCT

On multivariate logistic regression analysis, the parameters onset timing, number of fine crackles in the inspiratory phase, F99 value, LDW, and age were independently associated with honeycombing in HRCT (Table 3). Their odds ratios (ORs) with 95% CIs were as follows: onset timing, OR 10.407 (95% CI, 1.366–79.298, *P* = 0.024); F99 value (unit Hz = 100), OR 5.953 (95% CI, 1.221–28.317, *P* = 0.029); number of fine crackles in the inspiratory phase (unit number = 5), OR 4.256 (95% CI, 1.098–16.507, *P* = 0.036); LDW, OR 1.337 (95% CI, 0.214–8.365, *P* = 0.756); and age, OR 1.074 (95% CI, 0.940–1.228, *P* = 0.293).

**Table 3:** Multivariate logistic regression analysis on acoustic features of fine crackles toward the presence of honeycombing findings

Variable	OR	95% CI	*P*
Timing (early vs. not early)	10.407	1.366–79.298	0.024
Frequency F99 (per 100 Hz)	5.953	1.221–28.317	0.029
Number of crackles (per 5 crackles)	4.256	1.098–16.507	0.036
LDW	1.337	0.214–8.365	0.756
Age	1.074	0.940–1.228	0.293

Data are presented as odds ratios (*OR*) with 95% confidence intervals (*CI*) and *P* values. The number of fine crackles was determined per inspiratory phase. *F99* Frequency in Hz at 99% of the total signal power, *LDW* Largest deflection width

**Fig. 2:**
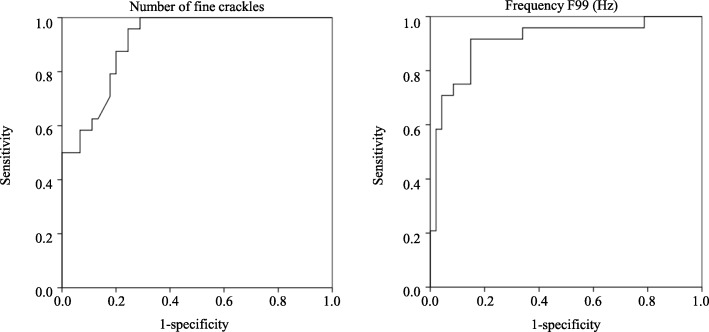
Receiver operating characteristic (ROC) curves for the number of crackles and the frequency F99 in patients with and without honeycombing. Area under the ROC curve: number of fine crackles, 0.913; frequency F99, 0.911

Subsequently, ROC curves were created for the continuous variables extracted by the multivariate logistic regression analysis, i.e., the number of fine crackles in the inspiratory phase and the F99 value (Fig. 2). The cutoff values to discriminate between the honeycombing and the non-honeycombing groups were for the number of fine crackles in the inspiratory phase 13.2 (area under the ROC curve [AUC], 0.913; sensitivity, 95.8%; specificity, 75.6%) and for the F99 value 752 Hz (AUC, 0.911; sensitivity, 91.7%; specificity, 85.2%). The multivariate logistic regression analysis using these cutoff values demonstrated that the odds ratio for the number of fine crackles in the inspiratory phase ≧13.2 was 33.907, whereas the odds ratio for the F99 value ≧752 Hz was 19.397 (Table 4).

**Table 4:** Multivariate logistic regression analysis using the following variables: number of crackles ≧13.2/inspiratory phase, onset timing (early), and F99 ≧752 Hz toward the presence of honeycombing findings

Variable	OR	95% CI	*P*
Number of crackles (≧13.2/inspiratory phase)	33.907	2.576–446.337	0.007
Frequency F99 (≧752 Hz)	19.397	2.311–162.813	0.006
Onset timing (early)	12.383	1.443–106.293	0.022

Data are presented as odds ratios (*OR*) with 95% confidence intervals (*CI*) and *P* values. *F99* Frequency in Hz at 99% of the total signal power

## Discussion

In the present study, we found that the honeycombing group had 1) an earlier beginning of the fine crackles within the inspiratory phase, 2) a higher number of crackles per respiratory cycle, and 3) an increased F99 value in the inspiratory phase compared with the non-honeycombing group. All three factors were able to detect the presence of honeycombing and distinguish this group from the non-honeycombing group at the defined significance level. To our knowledge, this is the first study comprehensively evaluating the relationship between the acoustic characteristics of fine crackles and the presence of honeycombing on HRCT.

Although fine crackles are generally thought to be heard during mid- to late-inspiration [16, 17], in our honeycombing group, the percentage of crackles in the early inspiratory phase was increased, whereas fine crackles did not begin in the late inspiratory phase in this study group. Moreover, the number of fine crackles per inspiratory phase in the honeycombing group was significantly elevated in comparison to that in the non-honeycombing group (21.5 versus 8.0, *P* < 0.001). A previous study reported that the number of crackles in pulmonary fibrosis was 18/inspiratory phase [18]. To discriminate between the honeycombing and the non-honeycombing group, the cutoff number of fine crackles in the inspiratory phase was determined as 13.2 with a sensitivity of 95.8% and a specificity of 75.6%. Furthermore, the number of crackles in the early phase was significantly increased in the honeycombing group in comparison to the non-honeycombing group (Table 2). This indicates that the earlier beginning of the fine crackles in the honeycombing group is not the reason for their increased number.

In our study, the power spectrum of fine crackles revealed in the honeycombing group an F99 value of 841 Hz, which was significantly higher than that in the non-honeycombing group (689 Hz). This F99 value in the honeycombing group was almost equivalent to the maximum frequency in pulmonary fibrosis (908 Hz) and the maximum frequency in IPF (721 Hz) which are diseases frequently characterized by honeycomb lungs [19, 20]. Ono et al. reported that the F50 and F75 values in an interstitial pneumonia group were positively correlated with the fibrosis score in HRCT (*r* = 0.783, *P* < 0.001 and *r* = 0.759, *P* < 0.001, respectively) [21]. Likewise, we showed that the pitch of fine crackles, known to be higher than that of coarse crackles, was increased especially in the honeycombing group. Our cutoff value (F99 value, 752 Hz) based on a ROC curve analysis was able to deduce the presence of honeycombing on HRCT with a sensitivity of 91.7% and a specificity of 85.2%.

The combined measurements of 2CD and IDW [14] with LDW [22] were reported to be useful for the discrimination between fine and coarse crackles. The CORSA guidelines, released in 1990, define the 2CD value of fine crackles as < 10 ms [9]. This value is commonly used in recent studies examining crackles. Comparing the two study groups regarding time-expanded waveform parameters, 2CD and LDW were significantly shorter in the honeycombing group (5.5 ms and 1.8 ms, respectively) than in the non-honeycombing group (7.0 ms and 2.0 ms, respectively). Our results are consistent with previous studies on fine crackles in pulmonary fibrosis, where the parameter 2CD was determined in the pulmonary fibrosis groups with 4.4–7.7 ms [19, 20, 23], which was invariably the shortest value among the respective comparison groups in these studies. In addition, Kawamura et al. showed that 2CD was significantly shorter in a group with honeycombing on HRCT (5.34 ms) than in a group without honeycombing (6.37 ms) [24].

As described above, our study is almost consitent with previous reports investigating fine crackles in ILD. We suggest that a detailed analysis of the acoustic characteristics can predict honeycombing in clinical settings. Fine crackles are reportedly produced by sudden airway openings during inspiration [25]. The acoustic properties of the fine crackles in our patients with honeycombing may reflect the following pathological characteristics of honeycombing: collapsing of multiple fibrotic alveoli, dilation of alveolar duct and lumen, often together with traction bronchiectasis and the dilatation of peripheral airspace due to the surrounding fibrosis [26].

This study has several limitations. First, we studied only patients with fine crackles in order to reveal the acoustic differences of fine crackles between the honeycombing and the non-honeycombing group. We did not investigate the respiratory sound of patients with honeycombing regardless of fine crackles. Therefore, our conclusion that the analysis of respiratory sound characteristics can identify honeycombing is limited to patients with fine crackles. Second, we did not evaluate the correlation between the extent of honeycombing and the acoustic properties of fine crackles and did not take into account the impact of the other HRCT findings, i.e., radiological findings such as traction bronchiectasis, reticulation, and ground-glass opacity. Third, the three raters of HRCT findings were from the same university. Therefore, in order to reduce the risk of misclassification bias, other raters from different institutions should be used in future studies. Fourth, the lung sounds were recorded on a particular site over the bilateral posterior base to standardize the recording method. However, it is possible that these recording sites are not the best for every patient. Thus, in future studies, improvements in recording methods for the evaluation of fine crackles are warranted.

## Conclusions

Our study revealed that the acoustic properties of fine crackles in the honeycombing group differ from those in the non-honeycombing group, which is consistent with the findings in previous studies on pulmonary fibrosis. Furthermore, the number of crackles, their onset timing, and their frequency at 99% of the maximum frequency (F99) were extracted as independent predictors for honeycombing. Thus, auscultation performed routinely in clinical settings combined with respiratory sound analysis may be useful to predict the presence of honeycombing.

## Data Availability

The datasets used and/or analysed during the current study are available from the corresponding author on reasonable request.

## Abbreviations

2CD: Two-cycle duration
AUC: Area under the ROC curve
CI: Confidence interval
CORSA: Computerized Respiratory Sound Analysis
CT: Computed tomography
DL_CO_: Diffusing capacity of the lungs for carbon monoxide
HRCT: Honeycombing on high-resolution computed tomography
IDW: Initial deflection width
ILD: Interstitial lung disease
IPF: Idiopathic pulmonary fibrosis
KL-6: Krebs von der Lungen-6
LDW: Largest deflection width
OR: Odds ratio
PaO_2_: Partial pressure of arterial oxygen
ROC: Receiver operating characteristic
SpA: Surfactant protein A
SpD: Surfactant protein D
UIP: Usual interstitial pneumonia
VC: Vital capacity
